# Recording of weight in electronic health records: an observational study in general practice

**DOI:** 10.1186/s12875-018-0863-x

**Published:** 2018-11-17

**Authors:** Lisa D. M. Verberne, Markus M. J. Nielen, Chantal J. Leemrijse, Robert A. Verheij, Roland D. Friele

**Affiliations:** 1 Netherlands Institute for Health Services Research (NIVEL), P.O. Box 1568, 3500 BN Utrecht, The Netherlands; 2 Tilburg School of Social and Behavioral Sciences, Tilburg University, Tranzo, P.O. Box 90153, 5000 LE Tilburg, The Netherlands

**Keywords:** Overweight, Obesity, Body weights and measures, Electronic health records, Primary health care

## Abstract

**Background:**

Routine weight recording in electronic health records (EHRs) could assist general practitioners (GPs) in the identification, prevention, and management of overweight patients. However, the extent to which weight management is embedded in general practice in the Netherlands has not been investigated. The purpose of this study was to evaluate the frequency of weight recording in general practice in the Netherlands for patients who self-reported as being overweight. The specific objectives of this study were to assess whether weight recording varied according to patient characteristics, and to determine the frequency of weight recording over time for patients with and without a chronic condition related to being overweight.

**Methods:**

Baseline data from the Occupational and Environmental Health Cohort Study (2012) were combined with data from EHRs of general practices (2012–2015). Data concerned 3446 self-reported overweight patients who visited their GP in 2012, and 1516 patients who visited their GP every year between 2012 and 2015. Logistic multilevel regression analyses were performed to identify associations between patient characteristics and weight recording.

**Results:**

In 2012, weight was recorded in the EHRs of a quarter of patients who self-reported as being overweight. Greater age, lower education level, higher self-reported body mass index, and the presence of diabetes mellitus, chronic obstructive pulmonary disease, and/or cardiovascular disorders were associated with higher rates of weight recording. The strongest association was found for diabetes mellitus (adjusted OR = 10.3; 95% CI [7.3, 14.5]). Between 2012 and 2015, 90% of patients with diabetes mellitus had at least one weight measurement recorded in their EHR. In the group of patients without a chronic condition related to being overweight, this percentage was 33%.

**Conclusions:**

Weight was frequently recorded for overweight patients with a chronic condition, for whom regular weight measurement is recommended in clinical guidelines, and for which weight recording is a performance indicator as part of the payment system. For younger patients and those without a chronic condition related to being overweight, weight was less frequently recorded. For these patients, routine recording of weight in EHRs deserves more attention, with the aim to support early recognition and treatment of overweight.

**Electronic supplementary material:**

The online version of this article (10.1186/s12875-018-0863-x) contains supplementary material, which is available to authorized users.

## Background

Overweight and obesity, in particular, is an important public health issue which is strongly associated with multimorbidity, as well as an increased workload for general practitioners (GPs) [1, 2]. In many European countries, the GP acts as a gatekeeper, representing the first healthcare professional to address patients’ health problems. Therefore, general practices are recognised to be a good starting point for the identification and subsequent prevention and management of overweight [2].

The use of electronic health records (EHRs) supports primary health care, as they contain complete and structured documentation of all relevant information on the health status of a patient [3, 4]. Routine recording of weight or body mass index (BMI) in EHRs could help GPs recognise and treat overweight patients, and therefore merits investigation.

Studies on EHRs in general practice have reported that BMI and/or weight recording are generally poor [5–10]. Most of these studies focused on primary healthcare in the UK, and showed that BMI and weight recording varied according to patient characteristics, and that recording slightly improved between the 1990s and 2000s [6–8]. The improvement over time was probably influenced by the publication of guidelines on obesity management, as well as the introduction of the Quality and Outcomes Framework (QOF) in the UK in 2004. As a result of the QOF, the reimbursement of GPs became dependent on a number of performance indicators, including recording of patient BMI [11].

In the Netherlands, clinical guidelines for the treatment of obesity in general practice were introduced in 2010. A bundled payment system was also introduced, meaning that health insurers pay a fixed fee to cover the entire primary healthcare needs of patients with diabetes mellitus, chronic obstructive pulmonary disease (COPD), and cardiovascular disorders. This bundled payment system obligates the primary healthcare professionals to provide the health insurance provider with performance indicators, including the proportion of patients with a recorded BMI [12].

Over recent years, there has been increased attention on the health impacts of being overweight. Therefore, weight recording in Dutch general practices is also expected to has increased over time, especially for patients with a chronic condition for whom a bundled payment system exists. However, a recent study of routinely recorded data from patients with COPD by Dutch general practices highlighted that BMI was recorded less frequently than expected [13].

The extent to which weight management is embedded in general practices in the Netherlands is currently unknown. Thus, the purpose of the present study was to assess weight recording in Dutch general practices for a group of patients who self-reported as being overweight. The primary aim was to assess the association between weight recording and patient characteristics. The secondary aim was to determine and compare the frequency of weight recording over time in patients with and without a chronic condition related to being overweight.

## Methods

### Study design

In this observational study, data from the EHRs of general practices in the Netherlands that participated in NIVEL Primary Care Database (NIVEL-PCD) were combined with data from the Occupational and Environmental Health Cohort Study (AMIGO study). Both cross-sectional and longitudinal analyses were applied.

The NIVEL-PCD comprises anonymised data from the EHRs of a representative sample (~ 10%) of all general practices in the Netherlands [14]. In general practice, EHRs are used by GPs and practice nurses to record information on consultations, diagnostic measurements, drug prescriptions, referrals, and morbidity according to the International Classification of Primary Care version 1 (ICPC-1).

The AMIGO study is a longitudinal study on the occupational and environmental determinants of disease and well-being. Participants for this study were recruited through 99 general practices that participated in the NIVEL-PCD in 2011 and 2012. All patients born between 1945 and 1981 who were registered at one of the 99 general practices were invited by their GP to participate in the AMIGO study. In total, 14,829 patients filled in the informed consent form and completed the baseline questionnaire between April 2011 and July 2012. The design of the AMIGO study has been described in more detail by Slottje et al. [15].

### Patient data

Information on the patients’ sex, year of birth, height, weight, level of education, smoking status, and alcohol consumption was obtained from the baseline questionnaire of the AMIGO study. Patient age was calculated as 2012 minus their year of birth. Information on GP consultations, diagnostic measurements, and morbidity over the period from 2012 to 2015 was obtained from the NIVEL-PCD.

### Study population

**Fig. 1:**
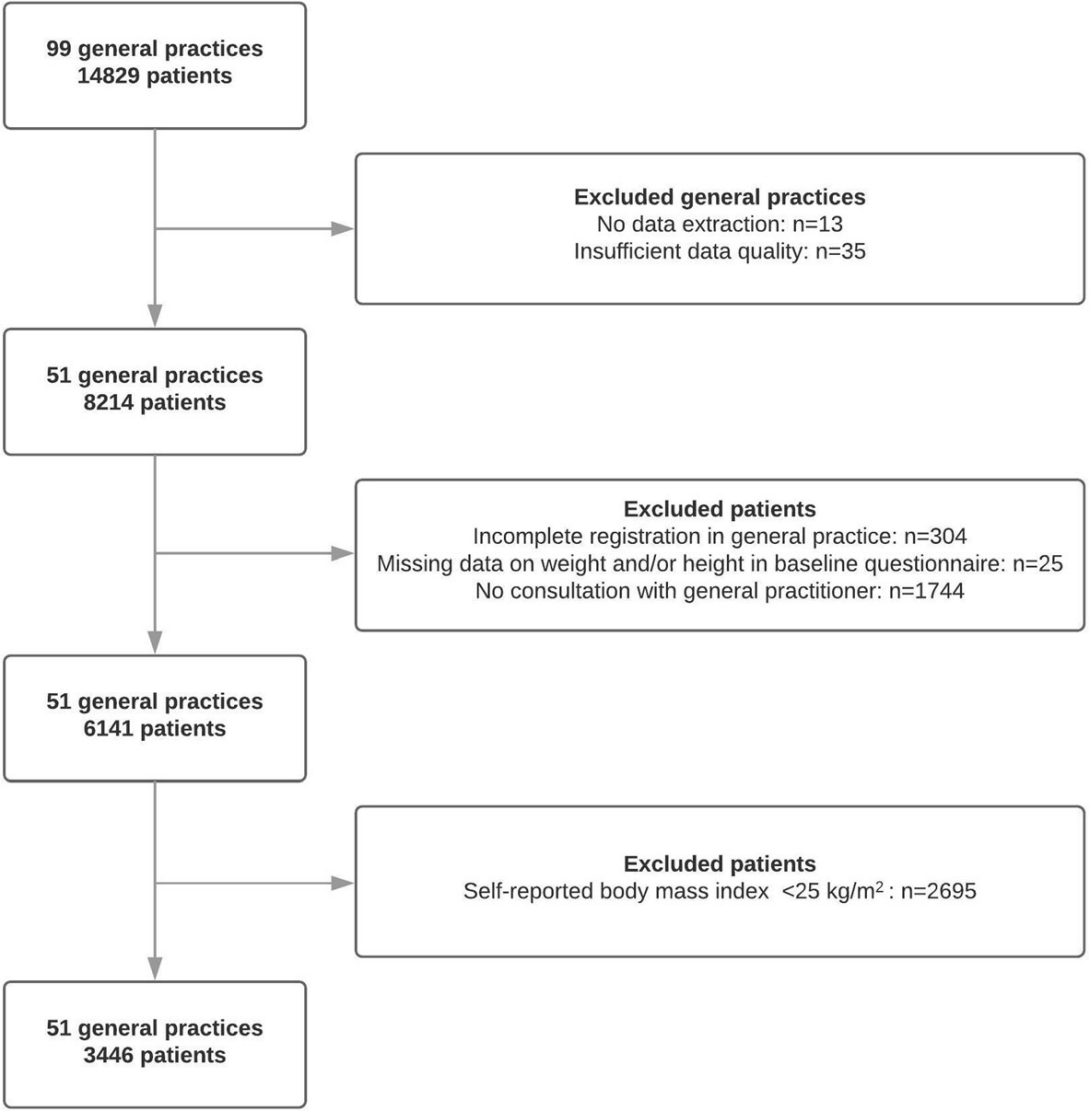
Selection process for the study population, 2012

Figure 1 shows the selection process for the study population included in the cross-sectional analyses, which used data from 2012. Eligibility criteria were applied at both the general practice and patient levels. Due to a failure in data extraction, data from 13 general practices that participated in the NIVEL-PCD were not available. Other practices (*n* = 35) were excluded due to poor data quality (i.e., data recording < 46 weeks/year, or < 70% of the recorded disease episodes labelled with the relevant ICPC code).

From the selected general practices, patients were excluded if they met the following criteria: (1) incomplete registration in general practice, (2) missing data on height and/or weight in the baseline questionnaire of the AMIGO study, or (3) no consultation with their GP in 2012 (because GPs needed to have the opportunity to record the weight of their patients). In total, 6141 patients from 51 general practices fulfilled these criteria, which appeared to be a representative sample of the total AMIGO study population (see Additional file 1). Subsequently, BMI was calculated using self-reported height and weight from the AMIGO study. A total of 3446 patients were classified as being overweight (i.e. BMI ≥25 kg/m^2^), who were included in the present study.

For the longitudinal analyses, data from 2012 to 2015 was used, and the eligibility criteria at the general practice level were also applied for the years 2013, 2014, and 2015. Furthermore, only patients who attended at least one annual GP consultation between 2012 and 2015 were selected. The final study population for the longitudinal analyses included 1516 patients.

### Outcome

For the cross-sectional analyses, a binary variable termed “weight recording” was generated, which indicated whether there was at least one BMI or weight measurement recorded in the patient’s EHR in 2012. For the longitudinal analyses, additional binary variables for “weight recording” were generated for the years 2013, 2014, and 2015.

### Independent variables

The following variables were generated from data recorded in the AMIGO study: BMI category (BMI ≥25 and < 30 kg/m^2^, BMI ≥30 kg/m^2^), sex (male, female), age (31–40 years, 41–50 years, 51–60 years, 61–67 years), highest achieved level of education (low, vocational education/community college; intermediate, vocational/high school; high, college/university or higher), smoking status (never, former, current), alcohol consumption (never, ≤1 day/week, 2–3 days/week, 4–5 days/week, 6–7 days/week). These variables were similarly categorised as presented in the design article for the AMIGO study [15].

Four (clusters of) chronic conditions known to be associated with overweight were selected based on morbidity data from the 2012 NIVEL-PCD: cardiovascular disorders (ICPC K74-K76, K86-K87, K89-K92, K99, T93), osteoarthritis (ICPC L89-L91), diabetes mellitus (ICPC T90), and COPD (ICPC R91, R95). We created an additional binary variable indicating whether a patient had none or at least one of the four selected (clusters of) chronic conditions related to being overweight. Furthermore, the mean BMI of each patient was calculated from all available recorded BMI (or height and weight) measures recorded in 2012.

### Statistical analyses

Descriptive statistics were used to present patient characteristics for 2012 and to determine the frequency of weight recording in the period from 2012 to 2015 for patients with a chronic condition related to being overweight (cardiovascular disorder, osteoarthritis, diabetes mellitus, and COPD), and for patients without a chronic condition related to being overweight. To assess which patient characteristics were associated with weight recording, univariate and multiple logistic multilevel regression analyses were conducted on the data from 2012. For the multiple regression analyses, two models were used. The first model included socio-demographic and lifestyle determinants (sex, age, education level, BMI category, smoking status, and alcohol consumption). In the second model, five variables related to the presence or absence of the four (clusters of) chronic conditions were added. A two-sided *P*-value < 0.05 was considered statistically significant, and the statistical analyses were performed with STATA 14.2.

## Results

Characteristics of the 3446 patients from data recorded in 2012 are presented in Table 1. Recordings of BMI (or height and weight) in EHRs were available for 23% (*n* = 805) of the patients. Of these 805 patients, 97% were also classified as being overweight according to their mean recorded BMI.

**Table 1:** Characteristics of the study population in 2012 (*N* = 3446)

		Number	Percent
Sex^a^	Male	1657	48.1
	Female	1789	51.9
Age category^a^	31–40 years	377	10.9
	41–50 years	954	27.7
	51–60 years	1260	36.6
	61–67 years	855	24.8
Education level^a^	Low	1224	36.6
	Intermediate	1116	33.4
	High	1002	30.0
BMI category^a^	≥25 & < 30 kg/m^2^	2380	69.1
	≥30 kg/m^2^	1066	30.9
Smoking status^a^	Never	1380	40.1
	Former	1528	44.4
	Current	532	15.5
Alcohol consumption^a^	Never	209	6.1
	≤ 1 day/week	1580	46.0
	2–3 days/week	734	21.4
	4–5 days/week	381	11.1
	6–7 days/week	533	15.5
Chronic condition^b^	Cardiovascular disorder	1461	42.4
	Osteoarthritis	343	10.0
	Diabetes mellitus	343	10.0
	COPD	145	4.2
Diagnostic measurements^b^	≥ 1 BMI record	756	21.9
	≥ 1 weight record	883	25.6
	≥ 1 height record	554	16.1

*BMI* body mass index, *COPD* chronic obstructive pulmonary disease

^a^Self-reported data (AMIGO-study)

^b^Data from electronic health records (NIVEL-PCD)

Table 2 shows the association of patient characteristics with weight recording for the 3446 patients who self-reported as being overweight in 2012. Greater age, lower education level, higher self-reported BMI, and the presence of a cardiovascular disorder, diabetes mellitus, or COPD were significantly associated with higher rates of weight recording in both univariate and multiple regression analyses. The strongest association was found for diabetes mellitus (adjusted OR = 10.3; 95% CI [7.3, 14.5]). The presence of a chronic condition related to being overweight was also strongly associated with age. The percentage of patients with at least one chronic condition related to being overweight increased from 3% in patients aged 31–40 years to 40% in patients aged 51–67 years.

**Table 2:** Association between patient characteristics and weight recording in general practice for self-reported overweight patients, 2012

	Univariate regression		Multiple regression			
			Model 1		Model 2	
	odds ratio	*p*-value	odds ratio	*p*-value	odds ratio	*p*-value
Sex^a^						
Male	Ref.		Ref.		Ref.	
Female	0.91 (0.77–1.06)	0.21	0.86 (0.73–1.03)	0.10	1.11 (0.91–1.35)	0.31
Age category^a^						
31–40 years	Ref.		Ref.		Ref.	
41–50 years	3.06 (1.95–4.81)	< 0.001	2.91 (1.84–4.60)	< 0.001	1.81 (1.10–3.00)	0.02
51–60 years	6.11 (3.95–9.47)	< 0.001	5.61 (3.59–8.75)	< 0.001	2.26 (1.38–3.72)	0.001
61–67 years	11.13 (7.15–17.32)	< 0.001	10.51 (6.66–16.58)	< 0.001	2.53 (1.51–4.23)	< 0.001
Education level^a^						
Low	Ref.		Ref.		Ref.	
Intermediate	0.62 (0.52–0.75)	< 0.001	0.81 (0.66–0.99)	0.04	0.83 (0.66–1.05)	0.12
High	0.52 (0.43–0.64)	< 0.001	0.63 (0.51–0.78)	< 0.001	0.70 (0.54–0.90)	0.005
BMI category^a^						
≥ 25 & < 30 kg/m^2^	Ref.		Ref.		Ref.	
≥ 30 kg/m^2^	1.77 (1.50–2.08)	< 0.001	1.77 (1.48–2.11)	< 0.001	1.25 (1.01–1.54)	0.04
Smoking status^a^						
Never	Ref.		Ref.		Ref.	
Former	1.43 (1.21–1.70)	< 0.001	1.11 (0.92–1.34)	0.27	1.08 (0.86–1.34)	0.52
Current	1.00 (0.78–1.28)	0.99	1.01 (0.78–1.32)	0.92	0.79 (0.58–1.08)	0.15
Alcohol consumption^a^						
Never	Ref.		Ref.		Ref.	
≤ 1 day/week	1.00 (0.72–1.41)	0.98	0.99 (0.69–1.44)	0.98	1.23 (0.80–1.90)	0.35
2–3 days/week	0.87 (0.61–1.25)	0.46	0.80 (0.54–1.20)	0.28	1.07 (0.67–1.72)	0.77
4–5 days/week	1.03 (0.70–1.53)	0.88	0.89 (0.58–1.37)	0.60	1.16 (0.70–1.93)	0.56
6–7 days/week	1.13 (0.78–1.63)	0.53	0.84 (0.55–1.28)	0.42	1.06 (0.65–1.74)	0.81
Chronic condition^b,c^						
Yes	Ref.		–	–	Ref.	
No	0.08 (0.07–0.10)	< 0.001	–	–	0.39 (0.25–0.60)	< 0.001
Cardiovascular disorder^b^			–	–		
No	Ref.		–	–	Ref.	
Yes	8.72 (7.23–10.51)	< 0.001	–	–	3.16 (2.16–4.62)	< 0.001
Osteoarthritis^b^			–	–		
No	Ref.		–	–	Ref.	
Yes	1.64 (1.28–2.09)	< 0.001	–	–	0.73 (0.53–1.00)	0.05
Diabetes mellitus^b^			–	–		
No	Ref.		–	–	Ref.	
Yes	18.34 (13.70–24.55)	< 0.001	–	–	10.27 (7.28–14.48)	< 0.001
COPD^b^			–	–		
No	Ref.		–	–	Ref.	
Yes	2.87 (2.03–4.05)	< 0.001	–	–	2.00 (1.31–3.06)	< 0.001

Odds ratios are presented with their 95% confidence interval

*BMI* body mass index, *COPD* chronic obstructive pulmonary disease

^a^Self-reported data (AMIGO-study)

^b^Data from electronic health records (NIVEL-PCD)

^c^Presence of cardiovascular disorder, and/or osteoarthritis, and/or diabetes mellitus, and/or COPD (yes/no)

For both univariate and multiple regression analyses a random intercept was included to account for clustered data of patients within general practices

Multiple regression analyses, model 1: sex, age, education level, BMI-category, smoking status, and alcohol consumption, model 2: model 1 + five variables related to the presence or absence of the four (clusters of) chronic conditions

In the period from 2012 to 2015, weight was recorded at least once for 58% of patients. Table 3 shows the frequency of weight recording over time for patients with and without a chronic condition related to being overweight. Weight was more frequently recorded for patients with diabetes mellitus. Between 2012 and 2015, 90% of patients with diabetes mellitus had at least one weight recording in their EHR, the majority (68%) of which had their weight recorded every year. For patients with a cardiovascular disorder or COPD, weight was recorded at least once for 80% of patients between 2012 and 2015. Weight was less often recorded for patients with osteoarthritis and for those without a chronic disorder related to being overweight. Between 2012 and 2015, 33% of patients without a chronic disorder related to being overweight had at least one weight measurement recorded in their EHR.

**Table 3:** Frequency of weight recording for self-reported overweight patients over the period from 2012 to 2015

	N	No weight recording (% patients)	At least one weight recording, but not annually (% patients)	Annual weight recording (% patients)
Patients with a cardiovascular disorder	730	20.1	46.2	33.7
Patients with osteoarthritis	167	34.7	47.3	18.0
Patients with diabetes mellitus	171	9.9	22.2	67.8
Patients with COPD	73	19.2	49.3	31.5
Patients without a weight related chronic disorder^a^	663	67.4	31.4	1.2

*COPD* chronic obstructive pulmonary disease

^a^ patients without a cardiovascular disorder, osteoarthritis, diabetes mellitus, and COPD

## Discussion

### Summary of findings

This study evaluated the extent of weight recording in general practices in the Netherlands among an adult population who self-reported as being overweight. Our findings show that greater age, lower education level, and higher self-reported BMI were positively related to weight recording. Furthermore, in accordance with our hypothesis, higher rates of weight recording were found for patients with diabetes mellitus, COPD, or cardiovascular disorders. These are all chronic conditions for which regular weight measurement is recommended in the clinical guidelines for GPs, and for which weight or BMI recording represents a performance indicator within a bundled payment system [12].

### Comparison with existing literature

The presence of diabetes mellitus was found to be the variable most strongly associated with weight recording, consistent with the findings of other studies [6, 9, 10, 16]. Furthermore, in line with a recent review of similar studies of the UK primary healthcare system, our study indicates that some patients are less likely to be identified as being overweight by their GP, including younger patients and patients without a chronic condition [17]. These findings are also supported by an Australian study which showed a positive association between age and weight recording [10], and a study of Dutch GPs’ weight management policy, which showed that weight was less often discussed with patients without weight-related comorbidities [18].

In contrast to other studies that indicate a higher frequency of weight recording in females, we found no difference between male and female patients. Furthermore, we found that a higher education level was associated with lower rates of weight recording, which differs from the results of a previous study which showed no association between education level and weight recording [16]. The discrepancy in findings related to socio-demographic characteristics may be due to differences in the selection criteria for study populations and the time-frames of studies, in addition to differences in healthcare systems between countries.

### Strengths and limitations

A strength of this study is the linkage of the cohort from the AMIGO study with routinely recorded data from general practices, which enabled us to combine information on self-reported socio-demographic and lifestyle determinants with health outcomes recorded in EHRs. Furthermore, to our knowledge, this is the first study to assess weight recording in Dutch general practices for patients who self-reported as being overweight.

A limitation of this study is the generalisability to the total population, as the study population consisted only of adults aged 31–67 years. Furthermore, we selected overweight patients based on self-reported height and weight, meaning that patients who did not identify themselves as being overweight were not included in the study. However, we do not believe that this had a large effect on the external validity of the study, as the proportion of overweight individuals (56%) in our study population is comparable to that of the general Dutch population of adults aged ≥20 years [19]. Additionally, our study showed good concordance between self-reported BMI and mean recorded BMI for patients with available data, so weight status does not seem to have been underestimated.

A representative sample size of the AMIGO study population was selected for the current study, even though it included only a subset of the cohort members. A large proportion of the initial study population had to be excluded due to insufficient data quality of the general practices, which might have resulted in a selection bias. The included general practices, which had higher levels of data quality, could potentially be systematically different to general practices with lower levels of data quality. However, we suspect that most of the variation in data quality among the general practices is unrelated to clinical performance, but instead due to software issues, as suggested by van der Bij and colleagues [4].

In the present study, we only included patients who had attended at least one consultation per year with their GP. Previous studies also only included ‘active’ patients, that is those who underwent a minimal number of consultations during a certain period [5, 9, 10]. Patients who do not consult their GP regularly are probably more healthy, and would therefore have their weight recorded by a GP less often. Thus, weight recording in our study population presumably occurred more frequently when compared to the total overweight population.

### Implications of findings

Routine weight recording in EHRs could help GPs identify overweight patients and monitor and support them in weight management programs, such as prevention programs that are embedded in primary healthcare [20, 21]. This study showed reasonable completeness of weight recording for overweight patients with a chronic condition, for whom regular weight evaluation is recommended in the clinical guidelines for GPs, and for which weight recording is incorporated as a performance indicator within a bundled payment system. In (relatively younger) overweight patients without a chronic condition related to being overweight, for whom weight measurement is not specifically required, we found that weight was recorded in only a third of these patients over a 4-year time frame. However, in the group of relatively young adults aged 31–40 years, the presence of overweight was already considerably high, with about 40% of patients classified as being overweight. To prevent weight-related health problems and their associated healthcare costs, discussing weight at an early stage should be recommended for this patient group.

Overweight management has been shown to be more frequent among patients with a documented weight status [22]. The importance of discussing overweight was illustrated by a large study conducted in the US, which found that patients were more likely to perceive themselves as being overweight, accompanied by an increased desire to lose weight, if they had been told by their healthcare professional they were overweight [23]. However, research from the UK suggests that GPs can feel uncomfortable talking about overweight, and may not always feel responsible for discussing weight management with their patients [24]. In the Netherlands, most GPs consider weight management to be part of their responsibility of providing care, but they face other barriers such as time constraints [18]. A solution may be for GPs to delegate some weight management tasks to practice nurses, who already play an important role in lifestyle counselling in Dutch primary healthcare [25]. In addition, providing feedback to general practices on their recording habits could help GPs become more aware of these habits, and would probably enhance weight recording [3, 4]. Furthermore, a performance indicator payment for weight recording in all patients could possibly improve recording and support early interventions in overweight individuals.

## Conclusions

This study of patients who self-reported as being overweight showed higher rates of weight recording in the EHRs of patients with a chronic condition, for whom regular weight measurement is recommended in the clinical guidelines for GPs, and for which weight recording is a performance indicator as part of a payment system. For younger patients and those without a chronic condition related to being overweight, weight was recorded considerably less often. For these patients, routine weight recording in EHRs deserves more attention in general practice, with the aim to support the early recognition and treatment of overweight individuals.

## Additional file

Additional file 1: Representativeness of study population. (DOCX 16 kb)

## Abbreviations

AMIGO study: Occupational and Environmental Health Cohort Study
BMI: Body mass index
COPD: Chronic obstructive pulmonary disease
EHR: Electronic health record
GP: General practitioner
ICPC-1: International Classification of Primary Care version 1
NIVEL-PCD: NIVEL Primary Care Database
QOF: Quality and outcomes framework
