# Supplementary material for: Recording of weight in electronic health records: an observational study in general practice
Source: BMC Fam Pract. 2018 Nov 17;19:174. doi: 10.1186/s12875-018-0863-x (PMC6240309; doi:10.1186/s12875-018-0863-x)
Supplement: Supplementary file 1 — Representativeness of study population. (DOCX 16 kb) [file 12875_2018_863_MOESM1_ESM.docx]

| **Representativeness of study population** | | | |
| --- | --- | --- | --- |
|  |  | **Study sample 2012** | **Total AMIGO population** |
| **Patients (N)** |  | 6141 | 14,829 |
| **Gender (%)** | Male | 42.4 | 44.3 |
|  | Female | 57.6 | 55.8 |
| **Age category (%)** | 31-40 years | 14.2 | 14.5 |
|  | 41-50 years | 28.9 | 29.3 |
|  | 51-60 years | 34.2 | 34.5 |
|  | 61-67 years | 22.7 | 21.8 |
| **BMI category (%)** | Underweight | 0.7 | 0.6 |
|  | Normal weight | 43.2 | 45.0 |
|  | Overweight | 38.8 | 38.7 |
|  | Obesity | 17.4 | 15.6 |
| **Education level (%)** | Low | 31.6 | 31.6 |
|  | Intermediate | 32.0 | 32.2 |
|  | High | 36.4 | 36.3 |
| **Smoking status (%)** | Never | 43.9 | 45.5 |
|  | Former | 39.4 | 38.8 |
|  | Current | 16.7 | 15.7 |
| **Alcohol consumption (%)** | Never | 5.7 | 5.7 |
|  | ≤ 1 day/week | 43.6 | 43.5 |
|  | 2-3 days/week | 22.2 | 22.4 |
|  | 4-5 days/week | 12.1 | 12.2 |
|  | 6-7 days/week | 16.5 | 16.2 |
